# Neuroepithelial Structures of the Oral Soft Tissues Including the Juxtaoral Organ of Chievitz: A Literature Review and Audit of Diagnosed Cases

**DOI:** 10.1007/s12105-020-01131-5

**Published:** 2020-02-03

**Authors:** Robert Kennedy

**Affiliations:** Guy’s and St Thomas’ NHS Foundation Trust, Oral/Head and Neck Pathology, King’s College London, Guy’s Hospital, 4th Floor Tower Wing, London, SE19RT UK

**Keywords:** Chievitz, Neuroepithelial, Oral

## Abstract

The juxtaoral organ of Chievitz (JOOC) is a part of microanatomy composed of bland epithelial islands closely associated with small nerves and usually described within the soft tissue on the lingual aspect of the posterior mandible. Similar structures are documented in the posterior tongue. There is a risk of misinterpretation as carcinoma showing perineural invasion. An audit was undertaken of diagnosed cases of the JOOC and similar neuroepithelial structures identified within the oral soft tissues of surgical specimens. Nineteen cases were identified. Epithelial islands ranged < 0.5–4 mm in maximum dimension and lay in close association with small nerves. Epithelial cells showed a squamoid appearance but were without keratinisation. There was no atypia and mitotic figures were not seen. In 53% of cases the epithelial cells showed cytoplasmic clearing, in 26% of cases there was brown pigment and in 11% of cases there were calcifications. In 53% of cases, these neuroepithelial structures lay within the soft tissue lingual to the mandible in the retromolar area, 26% of the structures were placed buccal to the mandible, 11% of the structures lay adjacent to the maxillary tuberosity and 11% of the structures were within the posterior tongue.

## Introduction

The juxtaoral organ of Chievitz (JOOC) is a part of normal microanatomy rarely encountered within resection specimens. It is a neuroepithelial structure composed of bland islands of epithelium in intimate association with nerve fibres and is described as located lingual to the angle of the mandible. While the structure’s physiological function remains obscure, its clinical importance arises from the risk of misinterpretation as carcinoma showing perineural invasion. The literature relating to neuroepithelial structures within the oral soft tissue is reviewed, and an audit of cases diagnosed at a single institute is presented.

## Materials and Methods

### Literature Review

The English language literature was surveyed to identify descriptions of neuroepithelial structures including the JOOC within the oral soft tissues in adults and children. The PubMed database was searched using the terms: JOOC; neuroepithelial structures AND oral. Cases without details of the anatomical site or without photomicrographs of interpretable quality were excluded. Cases with features suggestive of an alternate diagnosis were also excluded as detailed in the results section. All other cases were included.

### Audit

A text search was made of the Guy’s Hospital (London) oral pathology database using the terms Chievitz and neuroepithelial. The audit standard was set to be that the clinical presentation and microscopic appearances should be in keeping with published reports and therefore that diagnosis could be considered as correct. No immunohistochemistry was performed as part of the audit. In one case immunohistochemistry was available as it was undertaken at the time of reporting.

## Results

### Literature Review

Three series (more than one case included) of the JOOC (Table 1) were identified from the available English literature. Each involved targeted sampling of the soft tissue on the medial aspect of the angle of the mandible with the intent of finding the JOOC. The specimens utilised in these three series were surgical resections [1], cadavers and autopsy specimens [2, 3]. In total these studies examined 64 specimens and the JOOC was found in 34 of these. The JOOC was identified in males and females and in all age groups including neonates. The JOOC was described as small nests of squamoid cells in close association with small nerves. There was no keratinisation and no atypia. Cytoplasmic clearing and duct-like spaces nests were described in several cases. Melanin pigment was present in 1 case and calcification in 2 cases. The finding of the JOOC medial to the angle of the mandible and with these histological features is further supported by four single case reports [4–7] (Table 2).

**Table 1:** Published case series of the JOOC and other neuroepithelial structures

Site	Specimen type	No. of cases	Age Sex	Constant microscopic features	Inconstant microscopic features	Refs
Deep to medial pterygoid over angle of mandible	Autopsy specimens	14	26–84 years Sex not specified	Nests of bland squamoid cells close to small nerves No keratin formation	Clear cytoplasm in peripheral cells Lumen formation (1 case)	[3]
Medial to mandible in area of internal oblique ridge	Autopsy and cadaver specimens	11	Neonate-over 50 years Sex not specified	Nests of bland squamoid cells close to small nerves	Peripheral nuclear palisading Lumen formation Calcification (2 cases) Melanin pigment (1 case)	[2]
Medial to mandible where the ascending ramus joins the body	Carcinoma resection specimen	9	49–90 years 2 M:1F	Nests of bland squamoid cells close to small nerves No keratinisation	Intercellular bridges Lumen formation (3 cases)	[1]
Posterior tongue	Incidental findings in biopsies (3 cases) Finding in a biopsy of a firm painful area (1 case)	4	59–68 years 2 M:1F	Islands of squamoid islands close to subepithelial nerve plexus of taste buds No keratinisation	Clear cytoplasm and intercellular bridges (1 case) Peripheral nuclear palisading and lumen formation (1 case)	[8]

**Table 2:** Published single case reports of the JOOC and other neuroepithelial structures

Site	Specimen type	Age Sex	Microscopic features	Refs
Retromolar trigone	Carcinoma resection	50 F	Nests of bland squamoid cells close to small nerves Foci of calcification	[4]
Retromolar trigone	Carcinoma resection	46 F	Nests of bland squamoid cells close to small nerves Some cells show clear cytoplasm	[5]
Medial aspect angle of mandible	Carcinoma resection	35 M	Nests of squamoid cells closely placed to small nerves and resembling Pacinian corpuscles	[6]
Medial pterygoid region	Carcinoma resection	44 F	Nests of bland squamoid cells close to small nerves	[7]
Lingual gingivae lower molar region	Biopsy of firm irritating area	24 F	Nests of bland squamoid cells close to small nerves Areas of degeneration resulted in microcystic changes The structure was described as a hamartoma	[10]
Base of tongue	Biopsy of described slight abnormality	39 M	Nests of bland squamoid cells close to small nerves	[9]

There is a single case report of the JOOC on the medial of the mandibular angle showing structures resembling Pacinian corpuscles. These were described as ovoid-to-spherical lamellar structures demonstrating an inner core immunoreactive for neurofilament protein and outer core of flattened cells positive for epithelial membrane antigen [6].

Immunohistochemistry studies of the JOOC are otherwise very limited [7]. The epithelial cells of the JOOC are shown to label with a pancytokeratin antibody (AE1/3). Central epithelial cells are positive for CK19 and peripheral basal cells are positive for high molecular-weight keratin (34bE12). There is no expression of CK7, CK20 or S100 and the Ki-67 labelling index is < 1%.

Palazzolo et al. [8] published a series of 4 cases showing neuroepithelial structures in the posterior tongue appearing as squamoid islands without keratinisation and lying subjacent to the surface epithelium. Clear cytoplasm and intercellular bridges were present in one of the cases. In another case, peripheral palisading and focal areas of cystic degeneration or duct-like structures were observed. The islands lay in close association with the subepithelial nerve plexus of taste buds. The patients were between 59 and 68 years of age with an even gender distribution. Three cases were incidental findings in biopsies undertaken in a search for a primary site of metastatic carcinoma. One case was identified in a biopsy of a firm area of the tongue base associated with pain. A further case report [9] describes a similar neuroepithelial structure also in the posterior tongue. The patient was a 39 year old male and the neuroepithelial structure was found in a biopsy taken from what was described as an area of slight abnormality.

A neuroepithelial hamartoma has been described in the lower molar lingual gingivae of a 24 year old female [10]. The report documents islands of squamoid epithelium within a collagenous stroma in close association with small nerves. In this case keratin pearl formation was seen focally in addition to microcysts. The descriptions were supported by good quality black and white photomicrographs, although the size of the hamartoma was not clear.

Exclusions from this literature review include reports of tumours of the JOOC that either did not include photomicrographs [11], included photomicrographs that were not of interpretable quality with for example nerves not being identifiable [12–14] or had features out of keeping with the JOOC including firm adherence to skin, keratotic pearls, isolated spindle cells and no reactivity with pancytokeratin immunostaining [15]. A hyperplasia of the JOOC was described in the bucco-temporal region in an autopsy specimen with no relevant history [16]. One of the islands was 4 mm in diameter and it was this that was designated as a hyperplasia. However, no nerves were described or could be identified in the photomicrographs and therefore the case was excluded.

### Audit

**Fig. 1:**
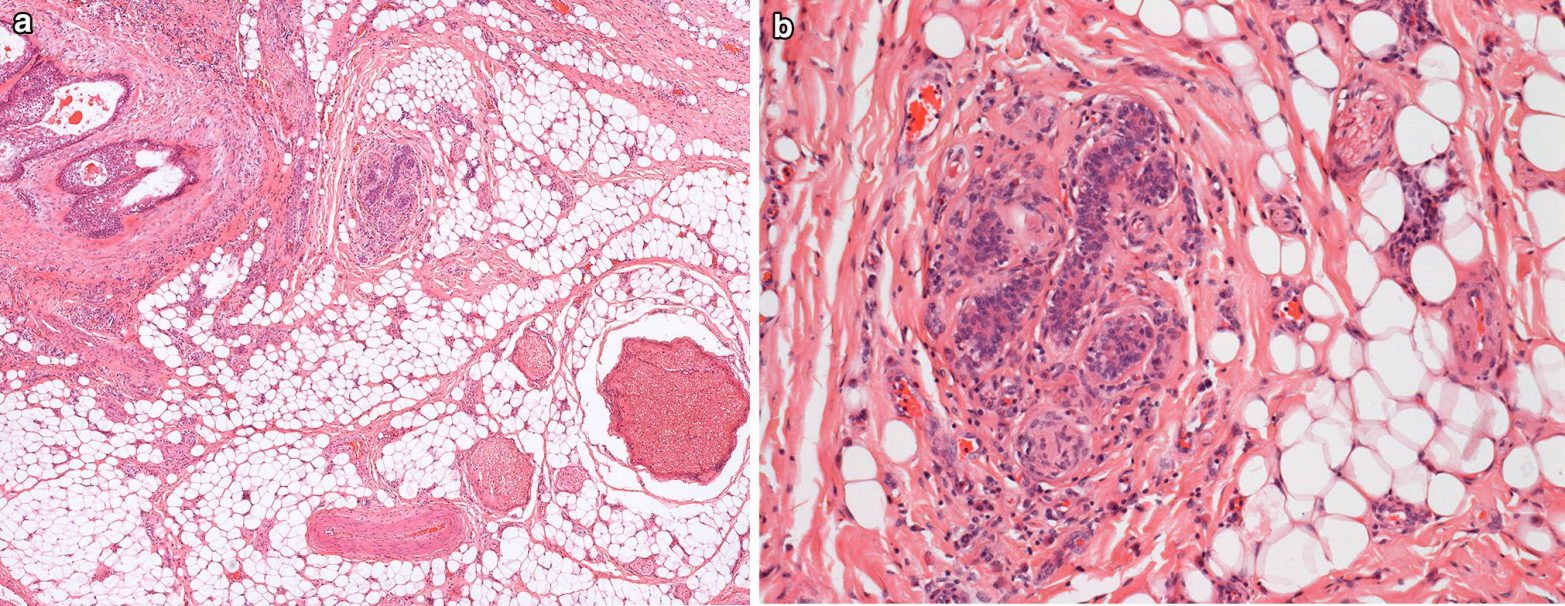
Section from a resection of a mucoepidermoid carcinoma (case 16). The site is buccal to the mandible and deep to muscle. **a** Shows the JOOC in the right of the field and the high grade mucoepidermoid carcinoma on the left (haematoxylin and eosin stain, × 10). **b** Shows the JOOC at high power (haematoxylin and eosin stain, × 20)

Nineteen diagnosed neuroepithelial structures were identified in the oral pathology archive (Table 3). The ages of patients demonstrating neuroepithelial structures including the JOOC ranged from 46 to 75 years old (mean age of 61 years). The male to female ratio was 2:1. Case 2 was an incidental finding in a resection of a clear cell odontogenic carcinoma. Case 3 was found within an excision from an area associated with pain on biting. Case 16 (Fig. 1) was an incidental finding in a resection of a mucoepidermoid carcinoma. Case 15 was present within a frozen section taken in the management of an osteosarcoma. The remaining cases were incidental findings in resections of squamous cell carcinoma.

**Table 3:** Audited cases of the JOOC and other neuroepithelial structures

	Site and demographics	Microscopic appearance	No. of epithelial islands	Max di
1	Medial to mandible Male, 7th decade of life	Squamoid islands within 0.5 mm of small nerves (< 0.5 mm in diameter) Brown pigment within fibrous tissue	4	< 0.5 mm
2	Medial to mandible, 8 mm below epithelium Deep to muscle Female, 5th decade of life	Squamoid islands within 0.5 mm of small nerves (< 0.5 mm in diameter)	1	0.5 mm
3	Medial to mandible Deep to muscle Male, 7th decade of life	Squamoid islands within 0.5 mm of small nerves (< 0.5 mm in diameter) Clear cytoplasm within peripheral cells	14	1.5 mm
4	Medial to mandible Deep to muscle Male, 8th decade of life	Squamoid islands within 0.5 mm of small nerves (< 0.5 mm in diameter) Clear cytoplasm within peripheral cells Brown pigment within fibrous tissue	4	0.5 mm
5	Medial to mandible Deep to muscle Male, 7th decade of life	Squamoid islands within 0.5 mm of small nerves (< 0.5 mm in diameter) Clear cytoplasm within peripheral cells	31	2 mm
6	Medial to mandible Deep to muscle Male, 8th decade of life	Squamoid islands within 0.5 mm of small nerves (< 0.5 mm in diameter) Central calcification	7	3.5 mm
7	11 mm medial to mandible Deep to muscle Deepest aspect level with inferior border of mandible Male, 8th decade of life	Squamoid islands within 0.5 mm of small nerves (< 0.5 mm in diameter) Concentric central calcification Brown pigment within fibrous tissue	9	4 mm
8	Medial to mandible 5.5 mm below epithelium Deep to muscle Female, 5th decade of life	Squamoid islands within 0.5 mm of small nerves (approximately 0.5 mm in diameter) Clear cytoplasm within peripheral cells Brown pigment within fibrous tissue	11	2.5 mm
9	Medial to mandible 9 mm below epithelium Deep to muscle Male, 6th decade of life	Squamoid islands within 0.5 mm of small nerves (approximately 0.5 mm in diameter)	5	< 0.5
10	Medial to mandible (retromolar region) 7 mm below epithelium Deep to muscle Male, 7th decade of life	Squamoid islands within 0.5 mm of small nerves (< 0.5 mm in diameter)	4	0.5 mm
11	Posterior tongue 1 mm below epithelium Within lamina propria of circumvallate papilla Male, 6th decade of life	Squamoid islands within 0.5 mm of small nerves (< 0.5 mm in diameter)	14	0.5 mm
12	Posterior tongue < 0.5 mm below epithelium Female, 6th decade of life	Squamoid islands close to taste buds	> 90	1.5 mm
13	Buccal to mandible 9 mm below epithelium Deep to muscle Female, 7th decade of life	Squamoid islands within 0.5 mm of small nerves (< 0.5 mm in diameter) Clear cytoplasm within central cells	3	< 0.5 mm
14	Buccal to mandible 7 mm below epithelium Deep to muscle Male, 7th decade of life	Squamoid islands within 0.5 mm of small nerves (< 0.5 mm in diameter) Clear cytoplasm within peripheral cells	4	0.5 mm
15	Buccal to mandible within submasseteric space Male, 6th decade of life	Squamoid islands within 0.5 mm of small nerves (< 0.5 mm in diameter) Clear cytoplasm within peripheral cells	1	< 0.5 mm
16	Buccal to mandible 6 mm below epithelium Deep to muscle Female, 6th decade of life	Squamoid islands within 0.5 mm of small nerves (< 0.5 mm in diameter)	14	0.5 mm
17	Buccal to mandible Deep to muscle Male, 7th decade of life	Squamoid islands within 0.5 mm of small nerves (< 0.5 mm in diameter) Clear cytoplasm within central and peripheral cells	3	0.5 mm
18	5 mm posterobuccal to maxillary tuberosity 4 mm below epithelium Deep to muscle Female, 6th decade of life	Squamoid islands within 0.5 mm of small nerves (< 0.5 mm in diameter) Clear cytoplasm within peripheral cells Brown pigment within fibrous tissue	2	0.5 mm
19	Superoposterior to maxillary tuberosity 5 mm below epithelium Deep to muscle Male, 7th decade of life	Squamoid islands within 0.5 mm of small nerves (< 0.5 mm in diameter) Clear cytoplasm within peripheral cells	7	0.5 mm

#### Anatomical Site

**Fig. 2:**
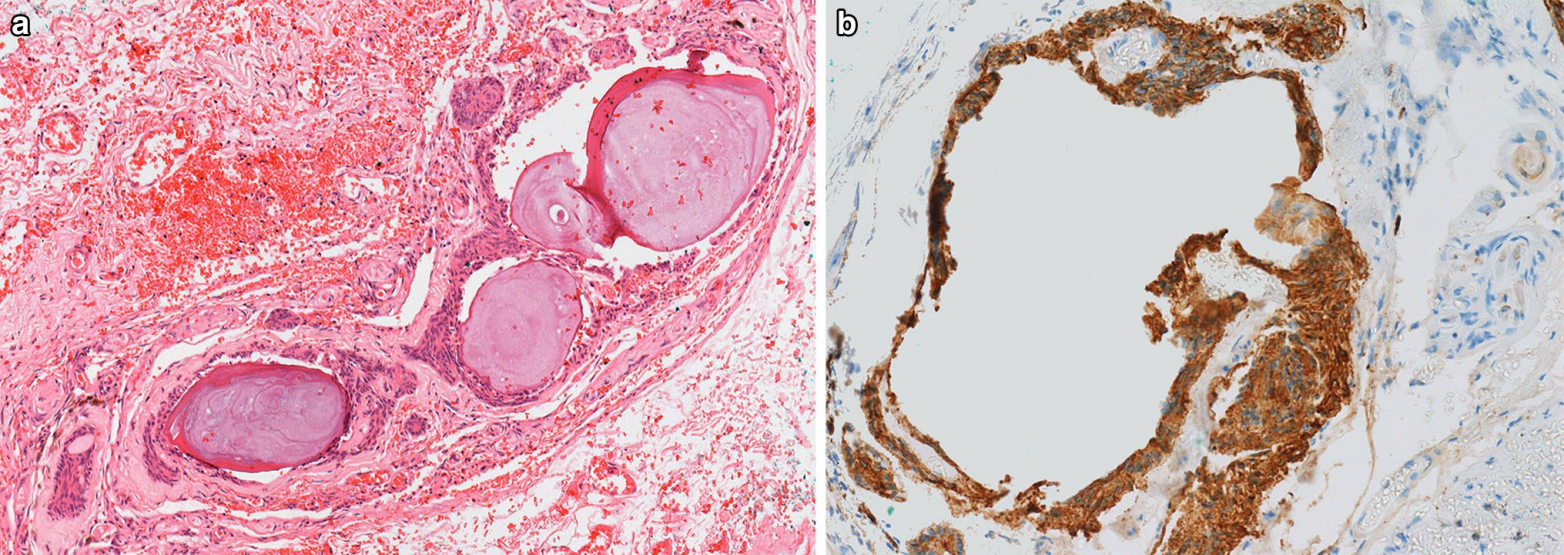
Section from a resection of a squamous cell carcinoma (case 7). The site is medial to the mandible and deep to muscle. This JOOC shows calcification and brown pigment on haematoxylin and eosin staining, × 10 (**a**). Immunohistochemistry for pancytokeratin (AE1/3) shows positive staining of the epithelium, the calcified area has fallen from the section resulting the defect seen (**b**)

**Fig. 3:**
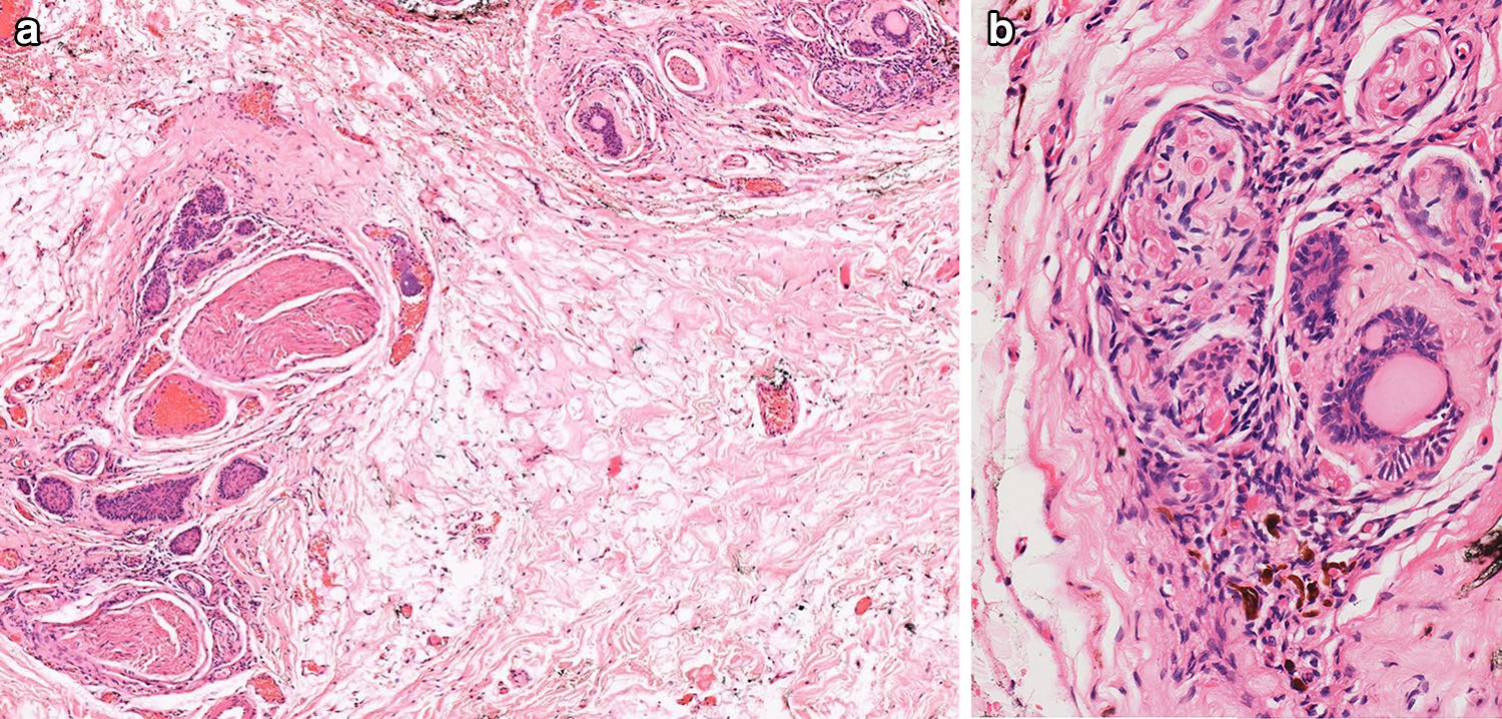
Section from a resection of a squamous cell carcinoma (case 8). The site is medial to the mandible. The JOOC comprises bland squamoid islands showing eosinophilic to focally clear cytoplasm. Brown pigment is also seen. Haematoxylin and eosin stain, × 10 (**a**) and × 20 (**b**)

**Fig. 4:**
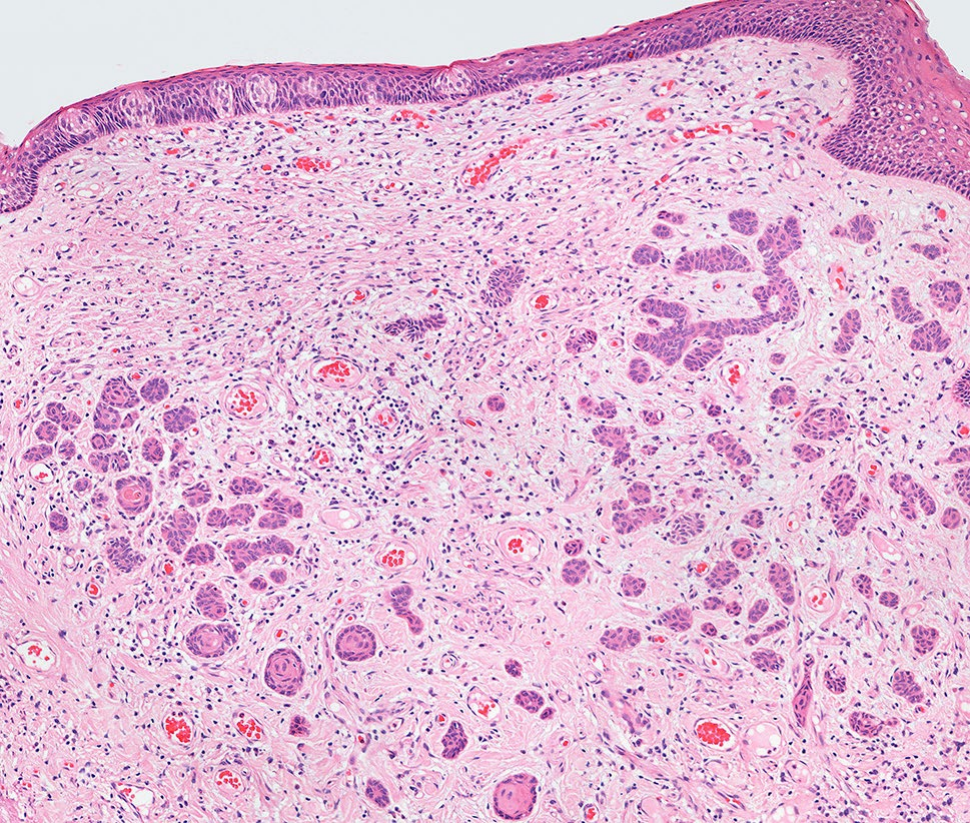
Section from a resection of a squamous cell carcinoma (case 12). The site is the posterior tongue. There are more than 90 squamoid islands closely placed to the taste buds. Haematoxylin and eosin stain, × 10

Ten cases (53%) were located within the soft tissue lingual to the mandible deep to muscle layers (Figs. 2 and 3). Five cases were described as buccal to the mandible (Fig. 1) and except for one case where assessment was not possible, were again localised below muscle. One of the cases buccal to the mandible (case 15) was a frozen section described as being taken from the submasseteric space and one of these cases (case 13) was an excision of the buccal mucosa that did not include the mandible. The remaining cases described as buccal to the mandible did include the mandible itself. Two cases were described as adjacent to the maxillary tuberosity and again lay below muscle. The precise relation of the location in these cases to the mandible was not clear. Two cases were in the posterior tongue and these lay within the lamina propria (Fig. 4).

#### Microscopic Appearance

**Fig. 5:**
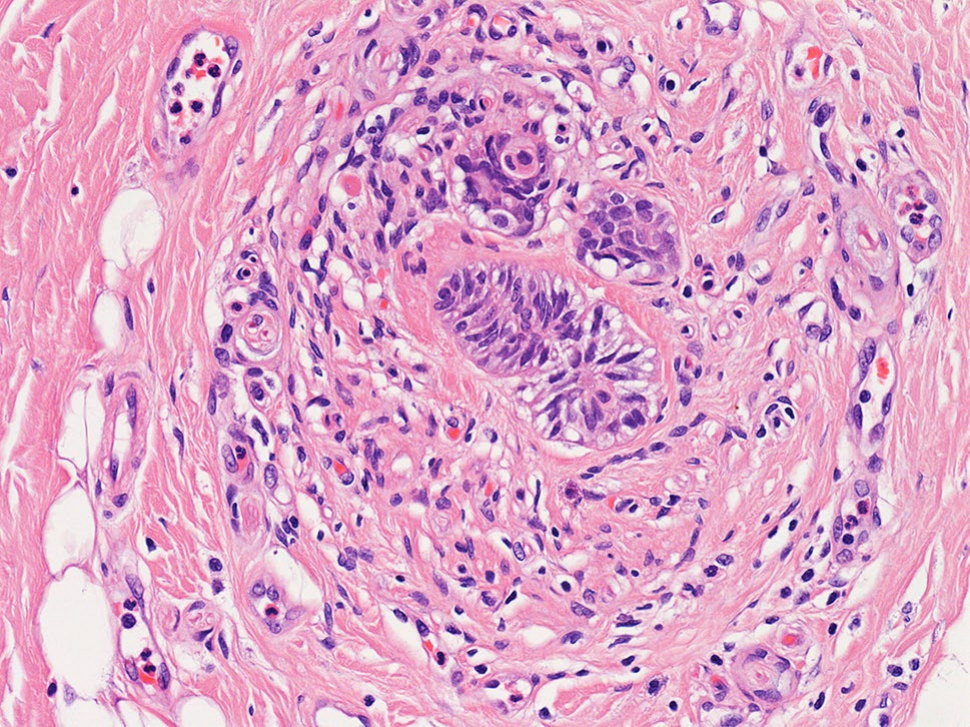
Section from a resection of a squamous cell carcinoma (case 13). The site is medial to the mandible. The JOOC comprises bland squamoid islands showing prominent cytoplasmic clearing and lying close to small nerves. Haematoxylin and eosin stain, × 20

In all cases the neuroepithelial structures appeared as well circumscribed rounded epithelial nests and islands numbering from 1 to more than 90 (Fig. 4). The maximum dimension ranged from < 0.5 mm (Fig. 5) to 4 mm (Fig. 2a). Islands and nests were located within fibrous tissue and lay within 0.5 mm of small nerves. In case 12 the neuroepithelial structure was observed in the posterior tongue and lay close to the taste buds (Fig. 4). There was a consistent squamoid appearance, the more central cells in the islands showing moderate amounts of usually eosinophilic cytoplasm and small rounded nuclei. The more peripheral cells often had a basal cell appearance with less abundant cytoplasm and slightly darker staining nuclei. There was no keratinisation and no mucin formation. There was no cytological atypia and no mitotic nor apoptotic figures.

Cytoplasmic clearing was present in 10 of the 19 cases (53%) (Fig. 3). Brown pigment was seen in 5 of the 19 cases (26%) (Figs. 2a, 3) and in one of these (case 7) immunohistochemistry had been undertaken at the time of reporting showing expression of cytokeratin (antibody to AE1/3) but no S100 expression, confirming an epithelial rather than melanocytic phenotype (Fig. 2b). Calcification was present in 2 cases (11%) (Fig. 2a). The cases in the posterior tongue showed no cytoplasmic clearing, no melanin pigment and no calcification (Fig. 4). In 7 cases there was an inflammatory infiltrate including neutrophils, lymphocytes and plasma cells. These were intermingled amongst the epithelial nests but in all cases, this was in keeping with a background of inflammation in surrounding areas. In no cases did the neuroepithelial structures themselves appear to attract an inflammatory response. Case 3 was a small specimen taken from an area associated with pain on biting. The specimen showed normal muscle, minor salivary glands and fibroadipose tissue within which was the neuroepithelial structure showing the same features as in the other cases. There was no inflammation and no evidence of any neural pathology.

## Discussion

All the cases in the audit showed neuroepithelial structures with microscopic features consistent with descriptions in the literature including a size up to 4 mm and an association with small nerves together with an absence of atypia and keratinisation. The audit standard of correct diagnosis was therefore met. Eighteen of the 19 cases were incidental findings within excision specimens as would be expected for a normal part of microanatomy. The finding of one case within a small excision from an area associated with pain on biting may also have been an incidental finding. It remains possible that some dysfunction of the neuroepithelial structure was the cause of the symptoms although the microscopic features were entirely typical and there was no evidence of inflammation. The most commonly described location was lingual to the mandible and this is in keeping with published cases. Two cases were described as adjacent to the maxillary tuberosity, although the relation of these sites to the mandible was not precisely clear. Five examples were described as buccal to the mandible but did show the typical histological features. The occurrence of neuroepithelial structures in the posterior tongue seen in two cases is previously documented. The largest previous series undertook targeted sampling of the tissues lingual to the mandible. The greater variation in anatomical location found in this audit is consistent with the cases being surgical resections rather than targeted samples.

In conclusion, neuroepithelial structures including the JOOC are most commonly located lingual to the mandible but can also be found buccal to the mandible and within the posterior tongue. The neuroepithelial structures appear as small (< 0.5–4 mm in maximum dimension) islands of squamoid epithelium in close association with small nerves or the taste buds. Cytoplasmic clearing, brown pigmentation and calcification are variably present. There is an absence of keratinisation, cytological atypia and mitotic activity. Attention to these details allows proper identification of benign neuroepithelial structures and avoidance of misinterpretation as carcinoma showing neural invasion.
